# Subtle Structural Modification of a Synthetic Cannabinoid Receptor Agonist Drastically Increases its Efficacy at the CB1 Receptor

**DOI:** 10.1021/acschemneuro.3c00530

**Published:** 2023-10-17

**Authors:** Hideaki Yano, Rezvan Chitsazi, Christopher Lucaj, Phuong Tran, Alexander F. Hoffman, Michael H. Baumann, Carl R. Lupica, Lei Shi

**Affiliations:** † Department of Pharmaceutical Sciences, School of Pharmacy and Pharmaceutical Sciences, Bouvé College of Health Sciences, Center for Drug Discovery, Northeastern University, Boston, Massachusetts 02115, United States; ‡ Computational Chemistry and Molecular Biophysics Section, National Institutes of Health, Baltimore, Maryland 21224, United States; § Electrophysiology Research Section, National Institutes of Health, Baltimore, Maryland 21224, United States; ∥ Designer Drug Research Unit, Intramural Research Program, National Institute on Drug Abuse, National Institutes of Health, Baltimore, Maryland 21224, United States

**Keywords:** synthetic cannabinoids, cannabinoid receptor 1, bioluminescence resonance energy transfer, molecular dynamics

## Abstract

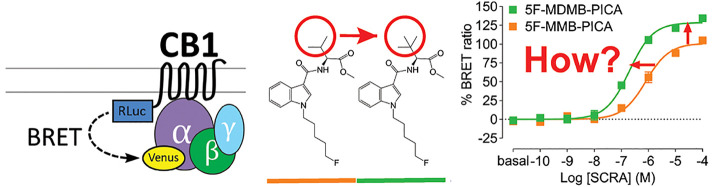

The emergence of synthetic cannabinoid receptor agonists (SCRAs) as illicit psychoactive substances has posed considerable public health risks, including fatalities. Many SCRAs exhibit much higher efficacy and potency compared with the phytocannabinoid Δ^9^-tetrahydrocannabinol (THC) at the cannabinoid receptor 1 (CB1R), leading to dramatic differences in signaling levels that can be toxic. In this study, we investigated the structure–activity relationships of aminoalkylindole SCRAs at CB1Rs, focusing on 5F-pentylindoles containing an amide linker attached to different head moieties. Using in vitro bioluminescence resonance energy transfer assays, we identified a few SCRAs exhibiting significantly higher efficacy in engaging the G_i_ protein and recruiting β-arrestin than the reference CB1R full agonist CP55940. Importantly, the extra methyl group on the head moiety of 5F-MDMB-PICA, as compared to that of 5F-MMB-PICA, led to a large increase in efficacy and potency at the CB1R. This pharmacological observation was supported by the functional effects of these SCRAs on glutamate field potentials recorded in hippocampal slices. Molecular modeling and simulations of the CB1R models bound with both of the SCRAs revealed critical structural determinants contributing to the higher efficacy of 5F-MDMB-PICA and how these subtle differences propagated to the receptor-G protein interface. Thus, we find that apparently minor structural changes in the head moiety of SCRAs can cause major changes in efficacy. Our results highlight the need for close monitoring of the structural modifications of newly emerging SCRAs and their potential for toxic drug responses in humans.

## Introduction

Synthetic cannabinoid receptor agonists (SCRAs) were originally developed as research compounds for studying the endocannabinoid system and subsequently repurposed as nausea suppressants, appetite stimulants, and for neuropathic pain relief.^1,2^ Unfortunately, since their introduction in medicinal chemistry, SCRAs have extensively infiltrated the recreational drug market as new psychoactive substances (NPS), leading to significant public health threats due to their elevated potential to cause psychiatric and medically serious disorders as well as mortality in humans. Over the past decade, the widespread use of these unregulated NPS has led to numerous cases of mass overdoses, accidental intoxications, and fatalities.^3,4^ Moreover, the increased efficacy and potency of SCRAs synthesized in illicit laboratories are associated with increased risks of their adverse and toxic effects.^5^ A striking example of the danger posed by SCRAs is a mass intoxication event that occurred in New York City in 2016, commonly termed a “zombie outbreak”. This event resulted from the use of the molecule MMB-FUBINACA, which resulted in symptoms including catatonia, seizures, and psychosis.^4^ Since this event, numerous additional reports have emerged describing deaths from the use of 5F-MDMB-PINACA.^3,6^ Where investigated, it was observed that these SCRAs exhibit high potency at cannabinoid receptors 1 and 2 (CB1Rs and CB2Rs), and they act as full agonists at CB1Rs. These features distinguish them from endogenous cannabinoids and the well-known psychoactive constituent of cannabis, THC, which are less potent and function as partial agonists.^7^

The CB1R, one of the most abundant G protein-coupled receptors (GPCRs) in the brain, primarily activates G_i/o_ proteins. This activation inhibits adenylyl cyclases, thereby reducing cAMP production and leading to the inhibition of gene transcription and synaptic remodeling.^8,9^ CB1Rs are widely expressed on axon terminals, where they can be activated by endocannabinoids. CB1Rs can also activate G protein-coupled inwardly rectifying potassium channels (GIRKs) to hyperpolarize neurons and inhibit voltage-gated calcium channels (VGCCs), primarily through the liberation of G protein β and γ subunits.^10−13^ The inhibition of neurotransmitter release by CB1Rs has been found in many neuronal pathways, including those mediated by glutamate and GABA, acetylcholine, and monoamines such as serotonin.^10−13^ In addition, CB1Rs expressed on astrocytes are reported to regulate the release of gliotransmitters that can further modulate neuronal activity.^14^ Together, through their ability to suppress neurotransmission in many neuronal circuits, CB1Rs and the endocannabinoid system regulate a wide variety of physiological functions, including learning and memory, motivation, pain, and anxiety.^9,15^

It is noteworthy that SCRAs induce a distinct subset of adverse effects such as nausea, anxiety, hypothermia, hallucination, catalepsy, tachycardia, hypertension, and withdrawal, which are unlike those associated with phytocannabinoids.^16^ Many SCRAs share an aminoalkylindole scaffold, which is divided into four connected moieties known as the “head, linker, core, and tail”.^1^ Structure–activity studies have identified several high-efficacy agonists at the CB1R, including 4CN-CUMYL-BUTINACA, 5F-MMB-PINACA, and 5F-CUMYL-PINACA, among others. All of these exhibit much higher potency and efficacy at CB1Rs compared to phytocannabinoids and many first-generation SCRAs, and this has been associated with much higher incidences of neurological symptomology.^17^ Additionally, some adverse effects of these SCRAs may also be attributed to off-site interactions with noncanonical receptor targets.^18^

Crystallographic and cryo-EM studies of CB1Rs have greatly advanced our mechanistic understanding of their function, revealing both active and inactive conformational states at the atomistic level.^19−24^ Notably, the active molecular structures bind to a variety of CB1R agonists, not only the widely used reference SCRA CP55940 but also a few other SCRAs such as AM11542, AM841, and MDMB-FUBINACA. Importantly, the active CB1R structures in complex with G_i_ protein can serve as excellent templates for investigating the structure-activity relationship (SAR) of SCRAs.

Synthetic orthosteric agonists that share binding sites yet elicit a higher maximum response than the endogenous agonist, resulting in an *E*_max_ greater than 100%, are considered superagonists for the target receptor.^25,26^ However, this concept is often contentious, as the readouts used to compare efficacies may be influenced by signal amplification, which leads to higher apparent *E*_max_ values. Despite the complexities of assay interpretation and the subject receptor system, there are instances where synthetic ligands indeed confer superagonism.^27,28^

5F-pentylindoles, a subgroup of highly potent and efficacious SCRAs, have been associated with a surge in human use and coinciding hospital visits.^16^ In this study, we aim to investigate the SAR of 5F-pentylindoles at CB1R, with a specific focus on comparing their “head” moieties that manifest varied efficacies. Our findings provide potential molecular mechanisms that may shed light on the superagonism observed at the CB1R.

## Results

### Subtle Changes in the Head Moiety of the 5F-Pentylindoles Result in Distinct Pharmacological Profiles of G_i1_ Engagement

Aminoalkylindole SCRAs, such as JWH018 and AM2201, which exhibit high potency and efficacy at the CB1Rs, were widely abused in the early 2010s.^4^ In this study, we investigated the SAR of the aminoalkylindole SCRAs with a 5F-pentylindole scaffold and focused on several molecules containing an amide linker attached to different head moieties. We first compared their functional properties in the G protein signaling associated with the CB1R. Specifically, to examine the coupling of CB1R and G_i1_ heterologously expressed in HEK293 cells, we employed a G protein engagement bioluminescence resonance energy transfer (BRET) assay.^29^ This method has the advantage over other functional assays such as adenylyl cyclase inhibition as it provides a direct measurement of G_i1_ coupling and is not confounded by potential off-target receptor activation of SCRAs. We collected readouts at four time points (2, 16, 30, and 44 min) of ligand incubation to capture kinetic profiles of receptor G-protein coupling, as lipophilic ligands are known to have binding kinetics different from hydrophilic ones.^30^ Results obtained at the 16 min time point were utilized in the analysis, as the responses of most agonists reached a stable plateau by this time point that did not further change.

**Figure 1:**
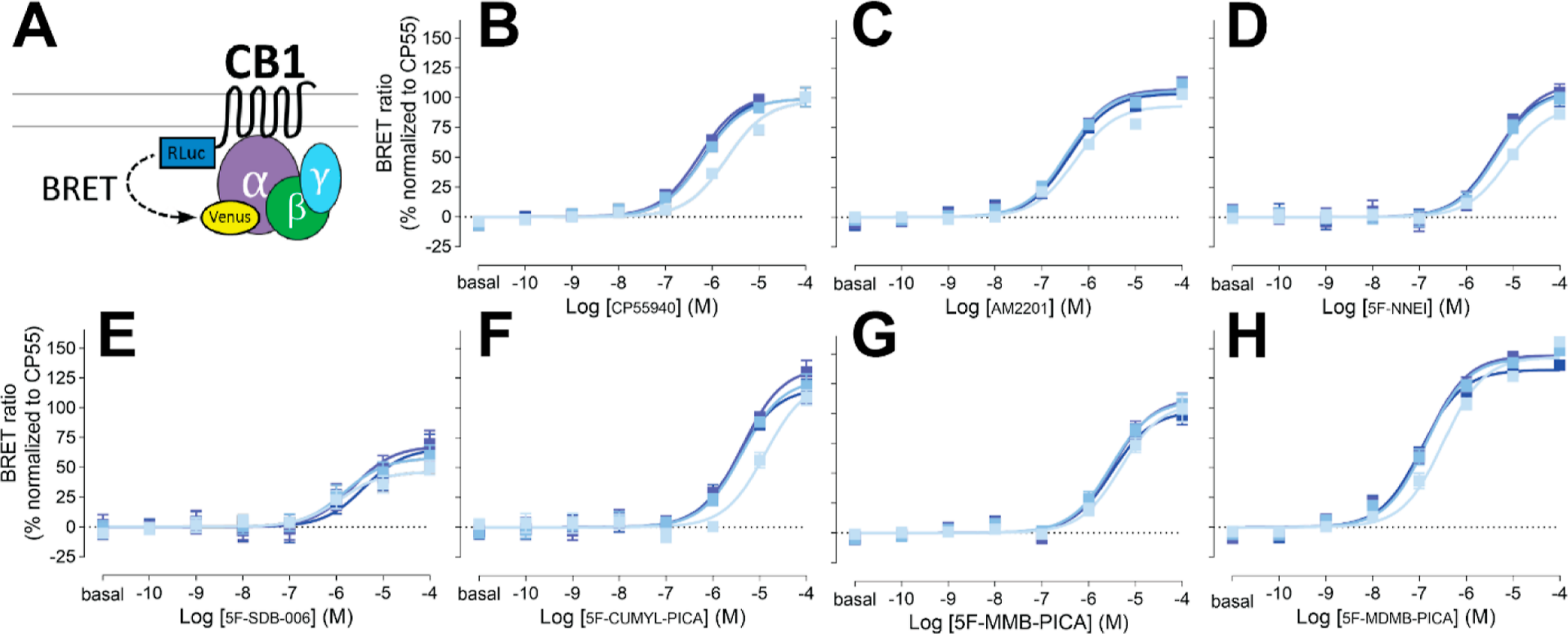
G_i1_ engagement BRET drug-induced engagement BRET between CB1R-Rluc and G_i1_-Venus (A) measured in response to CP55940 (B), AM2201 (C), 5F-NNEI (D), 5F-SDB-006 (E), 5F-CUMYL-PICA (F), 5F-MMB-PICA (G), and 5F-MDMB-PICA (H) at various time points (2, 16, 30, 44 min light to dark blue). Concentration–response curves are plotted as a percentage of maximal response by CP55940 at each time point and presented as means ± SEM of *n* ≥ 3 independent experiments.

Our results show that AM2201 displays a similar potency and efficacy profile to a commonly used reference full agonist, CP55940, with a slightly higher *E*_max_, although the difference is not significant (Table 1). Conversion of the ketone moiety of AM2201 to an amide linker results in a less potent compound (*N*-1-naphthyl, 5F-NNEI) compared to AM2201 (1-naphthyl) (see Figure 1, Supporting Information Figure S1, and Table 1). Substituting 1-naphthyl of 5F-NNEI’s head moiety with a less bulky benzyl group (*N*-1-benzyl and 5F-SDB-006) substantially lowers the efficacy but slightly increases the potency relative to 5F-NNEI.

**Table 1:** Pharmacological Comparison of 5-Fluoropentylindole Ligands at CB1R^a^

CB1R-G_i1_ engagement at 16 min											
	CP55940	AM2201	5F-NNEI	5F-SDB-006	5F-CUMYL-PICA	5F-MMB-PICA	5F-MDMB-PICA	WIN55-212-2	Δ^9^-THC	AEA	2AG
*E*_max_ (%)	100.0 ± 3.2	105.8 ± 2.3	106.2 ± 6.5	57.9 ± 6.2****	124.2 ± 6.7*	111.2 ± 5.2	142.1 ± 2.7****	98.8 ± 5.2	36.1 ± 4.1****^b^	57.8 ± 3.1****^b^	61.2 ± 2.7****^b^
pEC50 (M)	6.19 ± 0.08	6.47 ± 0.06	5.33 ± 0.12*	5.83 ± 0.25	5.38 ± 0.11	5.53 ± 0.10	6.85 ± 0.06	5.00 ± 0.09****	ND	4.92 ± 0.49	5.21 ± 0.16

CB1R-βarrestin 2 recruitment at 16 min											
	CP55940	AM2201	5F-NNEI	5F-SDB-006	5F-CUMYL-PICA	5F-MMB-PICA	5F-MDMB-PICA	WIN55-212-2	Δ^9^-THC	AEA	2AG
*E*_max_ (%)	100.0 ± 3.1	106.5 ± 1.9	110.3 ± 2.9	46.0 ± 2.5****	91.0 ± 3.8	99.9 ± 4.1	147.7 ± 1.6****	121.3 ± 4.7^b^	36.0 ± 7.1****^b^	49.9 ± 3.0****^b^	85.3 ± 4.9^b^
pEC50 (M)	5.19 ± 0.06	6.12 ± 0.04*	5.28 ± 0.05	5.61 ± 0.12	5.86 ± 0.10*	5.44 ± 0.09	6.24 ± 0.03*	4.72 ± 0.07	ND	4.95 ± 0.11	4.55 ± 0.92

a Mean ± SEM values for *E*_max_ and pEC_50_ are reported. *E*_max_ is normalized against CP55940.

b *Y*-axis cutoff value at the highest concentration is shown. ND—EC_50_ cannot be determined. One-way ANOVA followed by Tukey’s test *p < 0.05, ****p < 0.0001 against the corresponding CP55940 in the same assay.

Replacing the benzyl group of 5F-SDB-006 with a cumyl group, which effectively adds two methyl groups and results in 5F-CUMYL-PICA, substantially increased the *E*_max_ to 124% of that of reference CP55940. Substituting the benzyl group of 5F-SDB-006 with a methyl 3-methylbutanoate group, resulting in 5F-MMB-PICA, also increased the efficacy, which was comparable to 5F-NNEI. Most strikingly, adding an extra methyl group at the 3 position of 5F-MMB-PICA’s head moiety, resulting in 5F-MDMB-PICA, dramatically increased both the efficacy and potency of the compound. For comparison, we also included concentration response curves for additional structurally unrelated cannabinoid agonists (Supporting Information Figure S2 and Table 1). Among them, Δ^9^-THC, anandamide, and 2-arachidonoylglycerol showed lower potency and efficacy than CP55940.

### SAR for β-Arrestin 2 Recruitment by 5F-Pentylindoles is Similar to that for the G Protein Engagement

We then evaluated the functional properties of these 5F-pentylindole SCRAs in the β-arrestin pathway by carrying out an β-arrestin 2 recruitment BRET assay. β-Arrestins play a crucial role in both desensitizing GPCRs and initiating their own signaling cascades. Specifically, β-arrestin 2 is involved in CB1R desensitization and internalization.^31^ To ensure an unbiased comparison between the G_i1_ protein engagement and β-arrestin 2 recruitment in a heterologous system, the same CB1R Rluc-fusion construct used for the CB1R-G_i1_ coupling was also used for β-arrestin 2 recruitment (see Methods).

**Figure 2:**
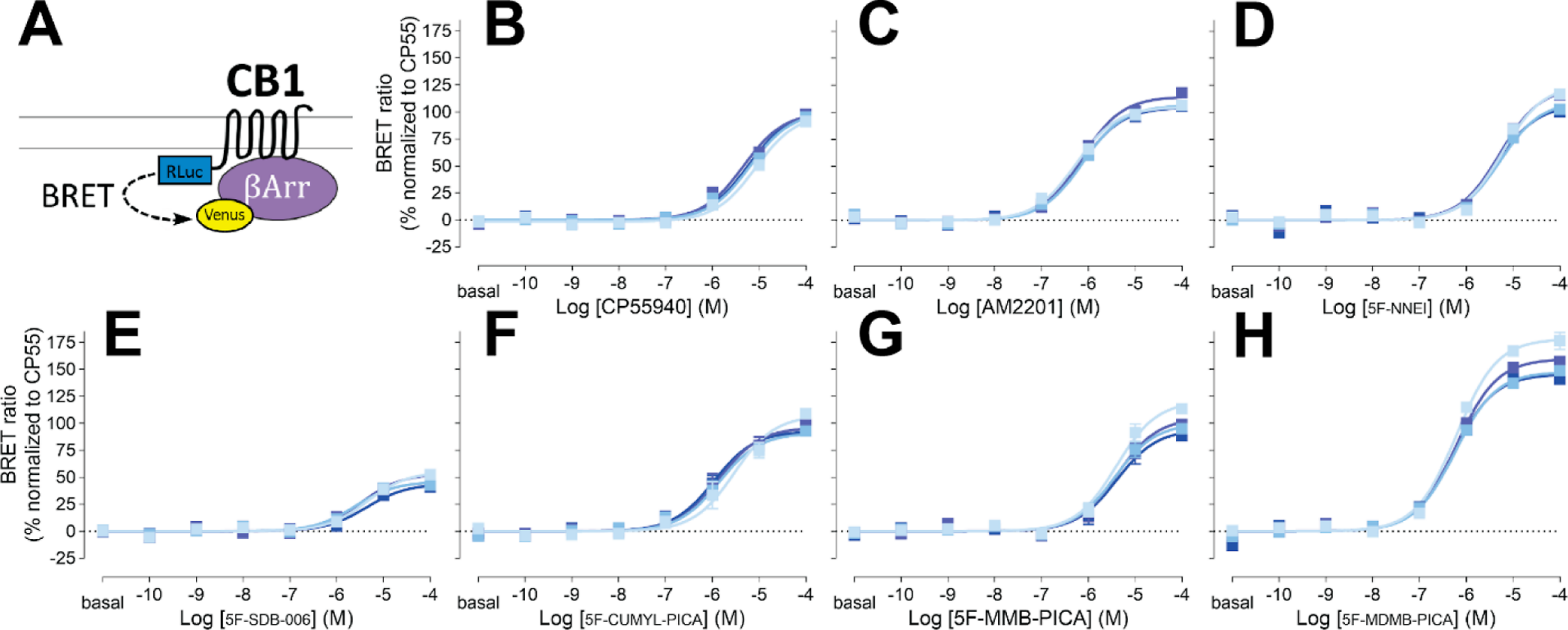
β-Arrestin 2 recruitment BRET drug-induced recruitment BRET between CB1R-Rluc and β-arrestin 2-Venus (A) measured in response to CP55940 (B), AM2201 (C), 5F-NNEI (D), 5F-SDB-006 (E), 5F-CUMYL-PICA (F), 5F-MMB-PICA (G), and 5F-MDMB-PICA (H) at various time points (2, 16, 30, and 44 min light to dark blue). Concentration–response curves are plotted as a percentage of maximal response by CP55940 at each time point and presented as means ± SEM of *n* ≥ 3 independent experiments.

It has been reported that CB1R agonists have lower potencies for β-arrestin recruitment compared to G protein coupling.^32^ As expected, most of the agonists tested in this study demonstrated ∼3-fold weaker potencies for β-arrestin 2 recruitment than for G_i1_ engagement (Figure 2 and Table 1). Nonetheless, the potency ranking among SCRAs for β-arrestin 2 recruitment was similar to that observed for G_i1_ engagement. Specifically, in both assays, the ketone-to-amide linker change resulted in decreased potency, as observed in the comparison between AM2201 and 5F-NNEI, while among all the tested 5F-pentylindoles with an amide linker, only 5F-MDMB-PICA showed a higher potency than AM2201. Furthermore, similar to that observed in the G_i1_ engagement assay, the addition of one extra methyl group from 5F-MMB-PICA to 5F-MDMB-PICA led to a more than 6-fold increase in potency. However, CP55940 noticeably showed a lower potency than all of the tested 5F-pentylindole SCRA in the β-arrestin 2 recruitment assay, which is not the situation in the G_i1_ protein engagement (Table 1).

For the efficacy of these SCRAs in recruiting β-arrestin 2, 5F-SDB-006 had the lowest efficacy among the tested 5F-pentylindoles, at only 46% of CP55940s efficacy, while 5F-MDMB-PICA showed a significantly higher efficacy than 5F-MMB-PICA, reaching 148% of CP55940s efficacy. Additionally, dose response profiles for other structurally unrelated cannabinoid agonists are displayed for comparison (Supporting Information Figure S3 and Table 1). Among them, THC, anandamide, and 2-arachidonoylglycerol showed lower potency and efficacy than CP55940.

### No Significant Bias was Observed between the G_i1_ Protein Engagement and β-Arrestin 2 Recruitment

**Figure 3:**
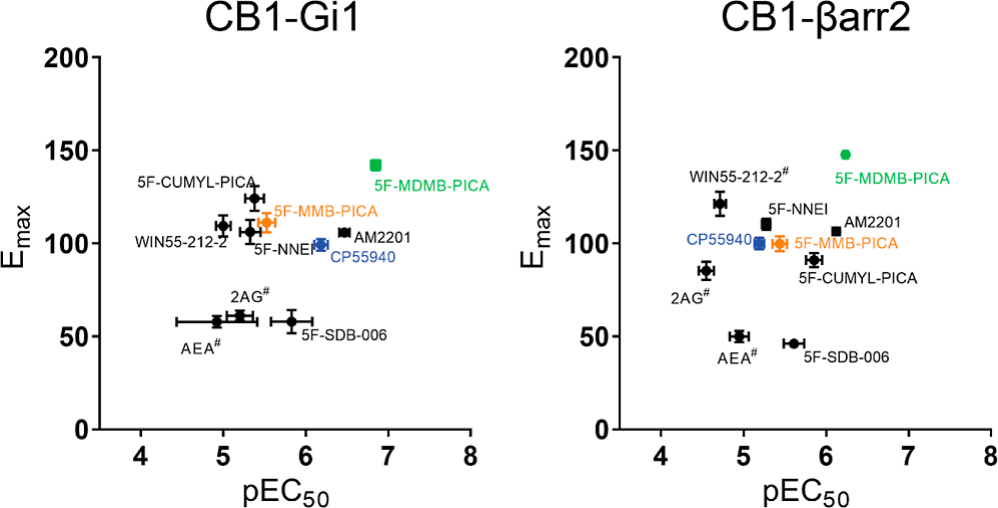
Tested SCRAs exhibited similar efficacy and potency trends in the G_i1_ protein engagement and β-arrestin 2 recruitment assays *E*_max_-pEC_50_ comparison between CB1R-G_i1_ engagement and β-arrestin 2 recruitment ^#^*Y*-axis cutoff value at the highest concentration is shown for its *E*_max_.

Signaling bias toward G protein activation or β-arrestin recruitment can result in downstream responses that deviate from those of balanced endogenous agonists. These differences could underlie the unique physiological outcomes reported for the use of SCRA in humans. We plotted the efficacies and potencies of various SCRAs and reference agonists for G_i1_ engagement and β-arrestin 2 recruitment (Figure 3). Many of the 5F-pentylindoles showed similar profiles for both signaling pathways, including 5F-MMB-PICA and 5F-MDMB-PICA (orange and green symbols in Figure 3). Notably, 5F-MDMB-PICA showed the highest efficacy and potency for both pathways. None of the agonists showed significant bias toward either pathway (i.e., with a bias factor >2.00 or <−2.00; Table 2).

**Table 2:** Coupling Bias between G_i1_ and β-Arrestin 2 among the Tested CB1R Agonists

bias factor	
CP55940	0.00
AM2201	–0.62
5F-NNEI	–0.97
5F-SDB-006	–0.68
5F-CUMYL-PICA	–1.34
5F-MMB-PICA	–0.86
5F-MDMB-PICA	–0.41
WIN55-212-2	–0.76

To further characterize the actions of SCRAs at the CB1R, the significant increase in 5F-MDMB-PICA’s efficacy compared to 5F-MMB-PICA in both assays, due to only one methyl difference in the head moiety, led us to focus on comparing these two SCRAs in subsequent ex vivo recording and molecular simulation studies.

### Significant Efficacy Difference was Observed between 5F-MDMB-PICA and 5F-MMB-PICA at Presynaptic CB1Rs in the Hippocampus

CB1Rs are expressed at the presynaptic terminals of neurons to regulate neurotransmitter release in the brain. In the hippocampus, CB1Rs are found on pyramidal neuron axonal terminals, known as Schaffer collateral/commissural (Scc) fibers projecting from the CA3 to CA1 regions.^33^ Activation of CB1Rs results in G_i/o_ protein-mediated inhibition of voltage-dependent Ca^2+^ channels, leading to a reduction in glutamate release from Scc fibers in CA1.^33,34^ We utilized extracellular recordings of the rising slopes of field excitatory postsynaptic potentials (fEPSPs), evoked by electrical stimulation of Scc fibers in hippocampal brain slices, to compare the effects of 5F-MDMB-PICA and 5F-MMB-PICA on glutamate release.^35^

**Figure 4:**
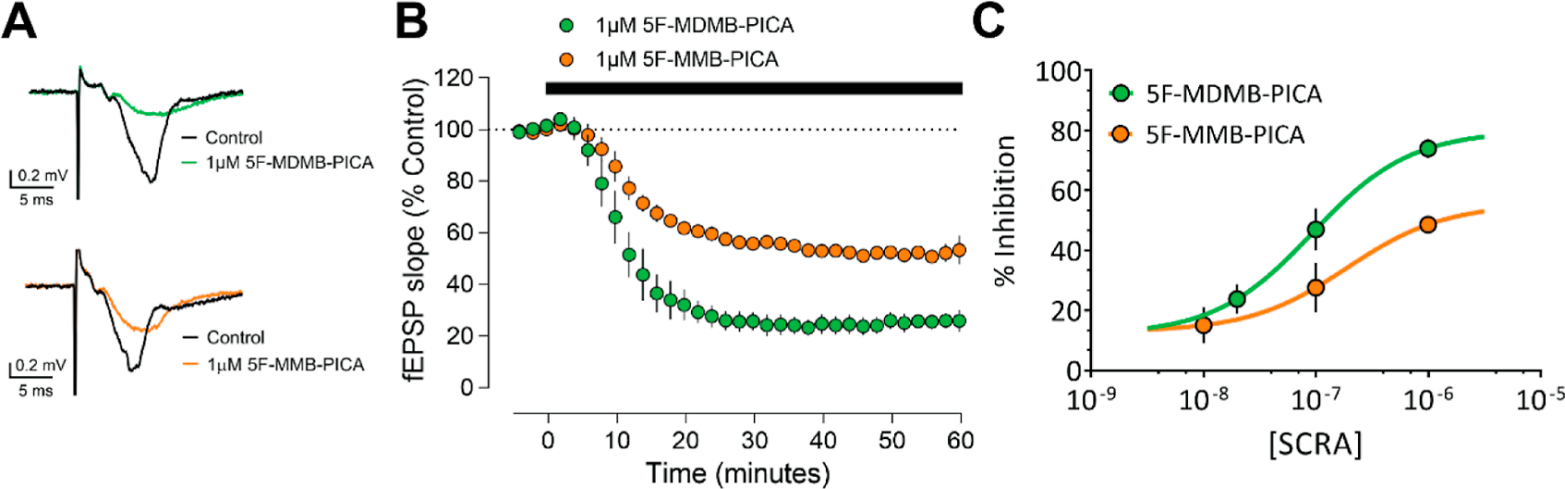
Brain slice electrophysiology concentration-dependent inhibition of hippocampal glutamate release by 5F-MDMB-PICA and 5F-MMB-PICA. (A) Representative averaged traces for 5F-MDMB-PICA (upper) and 5F-MMB-PICA (lower). (B) Summary time course of recordings (*n* ≥ 3 recordings) demonstrating the effect of SCRAs. (C) Concentration–response curve for 5F-MDMB-PICA and 5F-MMB-PICA (*n* ≥ 3 recordings per concentration). The pEC_50_ was calculated to be 7.20 and 6.72 for 5F-MDMB-PICA and 5F-MMB-PICA, respectively. Data points are presented as means ± SEM.

As previously demonstrated with other SCRAs,^36^ fEPSPs were significantly reduced by bath application of either 5F-MDMB-PICA or 5F-MMB-PICA (1 μM, Figure 4A). As depicted in a time-course plot of the fEPSP slope, the effects of both agonists are apparent within 10 min of application, with calculated tau values of 6.34 and 8.80 min for 5F-MDMB-PICA and 5F-MMB-PICA, respectively (Figure 4B). However, the maximal inhibition produced by 5F-MDMB-PICA was significantly greater than that observed with 5F-MMB-PICA. Thus, concentration–response curves revealed significantly different *E*_max_ values for the two SCRAs (Figure 4C, *t*-test *P* = 0.026). To validate the CB1R-dependence of these effects, we applied the CB1R-selective antagonist AM251 after the fEPSP reductions induced by the agonists were stable. We found that AM251 fully reversed the effects of both agonists, thus confirming that these effects were indeed mediated through CB1R (Supporting Information Figure S4).

### Adding an Extra Methyl Group to the Ligand Head Moiety Changes the Dynamics of the Agonist Binding Pocket

The in vitro and ex vivo assays demonstrated that 5F-MDMB-PICA is significantly more efficacious than 5F-MMB-PICA (Figure 3). The fact that only the addition of a single methyl group to the head moiety of 5F-MMB-PICA could cause such a large increase in 5F-MDMB-PICA’s efficacy prompted us to investigate how this affects ligand–receptor interactions and the propagation to the receptor/G-protein coupling interface. To do this, we conducted comparative molecular modeling and simulations of CB1R bound to each of these SCRAs.

**Figure 5:**
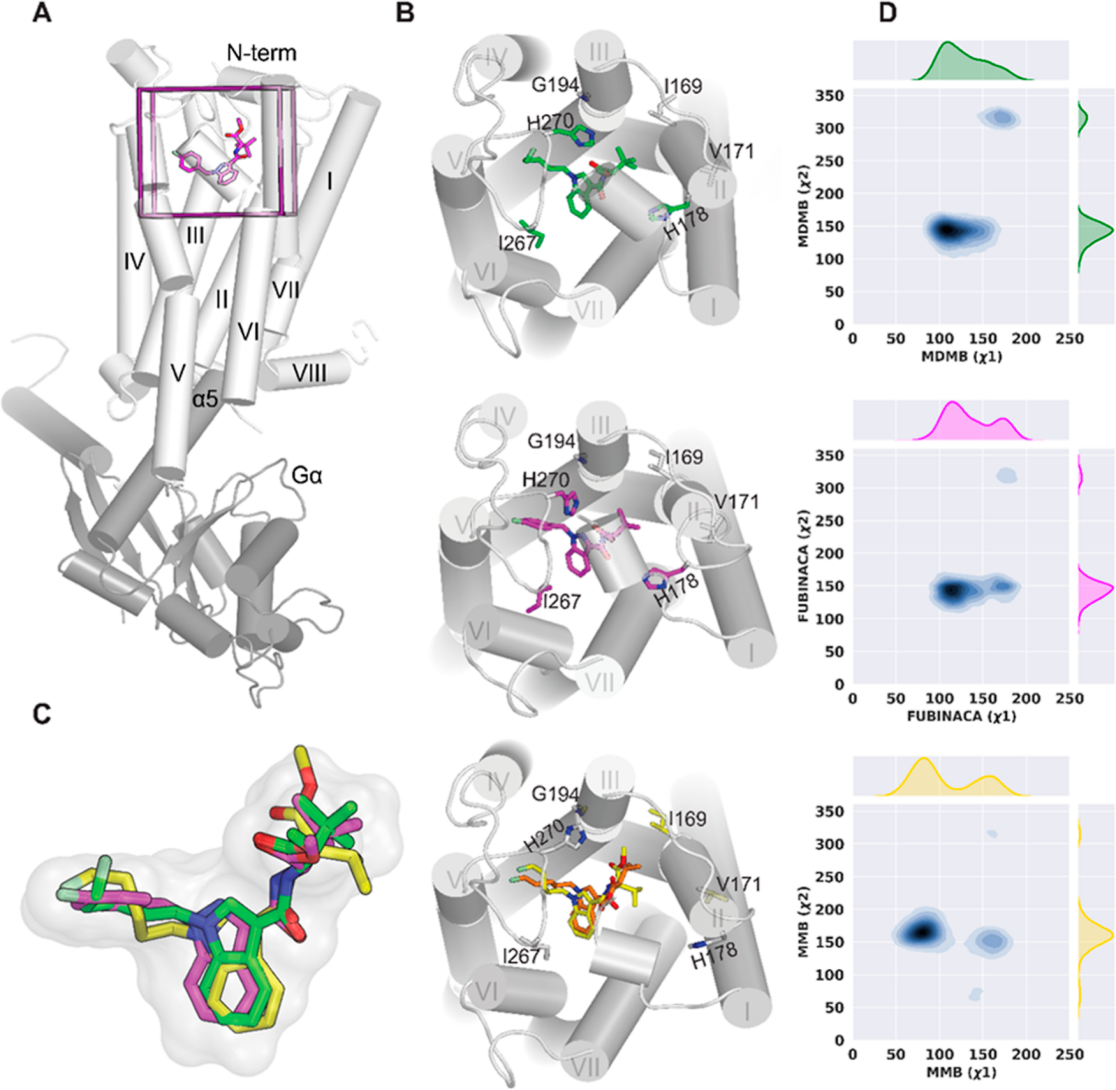
Ligands’ dihedral angle and binding pocket. (A) Zoom-out view of the receptor (gray) bound with Gαi (dark gray) and MDMB-FUBINACA (magenta). (B) Binding mode of 5F-MDMB-PICA (green), MDMB-FUBINACA (magenta), and 5F-MMB-PICA (yellow and orange) and their associated binding site residues that show differences in contact frequency between 5F-MDMB-PICA, MDMB-FUBINACA, and 5F-MMB-PICA. The residues that show differences are color-coded correspondingly. (C) Zoom-in view of the superimposed ligands in the binding site. They occupy the same region in the binding pocket, but 5F-MMB-PICA picks two distinct conformers for the head moiety. (D) 2D map of head moiety dihedral angles (Supporting Information Figure S1).

We used the CB1R/Gαi protein complex bound with MDMB-FUBINACA (Figure 5A; PDB 6N4B) as the template to construct the CB1R/5F-MDMB-PICA and CB1*R*/5F-MMB-PICA complex models (see Methods). In the structure of 6N4B, the head moiety and linker of MDMB-FUBINACA occupy a pocket that is enclosed by residues from transmembrane helices (TMs) 1, 2, and 7, while its *p*-fluorobenzyl tail is enclosed by TMs 3, 5, 6, and the second extracellular loop (ECL2). Notably, 5F-MDMB-PICA and MDMB-FUBINACA share the same head moiety, while 5F-MMB-PICA has one fewer methyl group than these two ligands (Supporting Information Figure S5). Therefore, we assumed that 5F-MDMB-PICA or 5F-MMB-PICA would have a head–tail orientation similar to that of MDMB-FUBINACA in the ligand binding pocket (Supporting Information Figure S5) and therefore selected their corresponding docking poses accordingly to construct the complex models. As a control, we also simulated the CB1R/MDMB-FUBINACA model (Table 3). During our simulations, 5F-MMB-PICA and 5F-MDMB-PICA maintained their binding poses in the same orientation as MDMB-FUBINACA (Figure 5C) but exhibited some variations in both the local interactions within the binding pocket and the resulting global receptor conformation.

**Table 3:** Summary of Simulated Conditions and Simulation Lengths

ligand	number of trajectories	length of trajectories (μs)
5F-MDMB-PICA	5	14.5
5F-MMB-PICA	6	14.5
MDMB-FUBINACA	3	6.6

Based on the equilibrated simulation results, we first assessed the impact of the extra methyl group on the ligand binding poses and measured two dihedral angles in the head moiety for each bound ligand (designated as ***x***1 and ***x***2, Supporting Information Figure S5). The distribution of ***x***1 revealed that 5F-MMB-PICA had two distinct modes in the binding site, with ***x***1 (∼50 ≤ peak 1 ≤ ∼115 and ∼135 ≤ peak 2 ≤ ∼180), resulting from the rotation of the head moiety around the N–C bond (Figure 5D). In contrast, there appeared to be minimal energy barriers for the head moieties of the bound 5F-MDMB-PICA and MDMB-FUBINACA to transition between these two modes. The ***x***2 dihedral angle did not exhibit any noticeable differences among the three ligands.

To test the hypothesis that the distinct dynamics of the ligand head moiety in the binding pocket may impact their interactions with the receptor, we calculated the contact frequency and residue side chain dihedral angles for each ligand (see Methods). Our results revealed that contact residues were present in TM2, TM3, ECL2, TM5, TM6, and TM7, while TM2 and TM3 had more significant roles in ligand binding interactions than other segments (Supporting Information Table S1). Notably, the contact frequency and side chain dihedral angle analyses suggested that 5F-MDMB-PICA and MDMB-FUBINACA, with the same head moiety, shared similarities for most of their contacting residues, and their differences with 5F-MMB-PICA were similar (Supporting Information Table S1). Specifically, several residues from TM2 (I169^2.56^, V171^2.58^, S173^2.60^, and H178^2.65^), TM3 (G194^3.30^), and ECL2 (I267^ECL2^, P269^ECL2^, and H270^ECL2^), which all interact with the ligand head moieties, showed similar differences.

In particular, our analysis shows that H178^2.65^ plays a crucial role in ligand interactions, with both 5F-MDMB-PICA and MDMB-FUBINACA interacting more frequently (∼100%) with this residue than did 5F-MMB-PICA (46%). In addition, our results reveal that 5F-MDMB-PICA and MDMB-FUBINACA interacts more with I267^ECL2^, I269^ECL2^, and/or H270^ECL2^ compared to 5F-MMB-PICA (Supporting Information Table S1 and Figure 5B). Furthermore, we observed that the ***x***1 rotamer of S173^2.60^ for 5F-MDMB-PICA and MDMB-FUBINCA has more than a 70° difference from that of 5F-MMB-PICA. Additionally, there are noticeable differences in the side chain conformation of P269^ECL2^ between 5F-MDMB-PICA/5F-MMB-PICA and MDMB-FUBINACA/5F-MMB-PICA (Supporting Information Table S1), which could be related to their different puckering states.

### Extra Methyl Group in Head Moiety Causes Conformational Changes in TM1 and TM2

To investigate the conformational changes beyond the ligand pocket, we first compared the available CB1R structures in the active and inactive states. The CB1R shares activation features with other class A GPCRs, including outward movement of the intracellular portion of TM6 (TM6i) and inward movement of TM7i, accompanied by changes in conserved motifs. Our quantitative analysis, shown in Supporting Information Figure S6, demonstrates these movements between the active CB1R-Gαi structures (PDB: 6N4B, 6KPG, and 7WV9)^20−22^ and the inactive structures (PDB: 5U09 and 5TGZ).^23,24^ In addition, CB1R also exhibits inward movements of the extracellular portions of TM1 (TM1e) and TM2e upon activation.^20^ TM2e plays a distinct role in CB1R activation by engaging the ligand in hydrophobic/polar interactions and stabilizing the agonist binding conformation.^20,24^ Interestingly, 6N4B shows an inward movement of TM6e as well (Supporting Information Figure S6).

**Figure 6:**
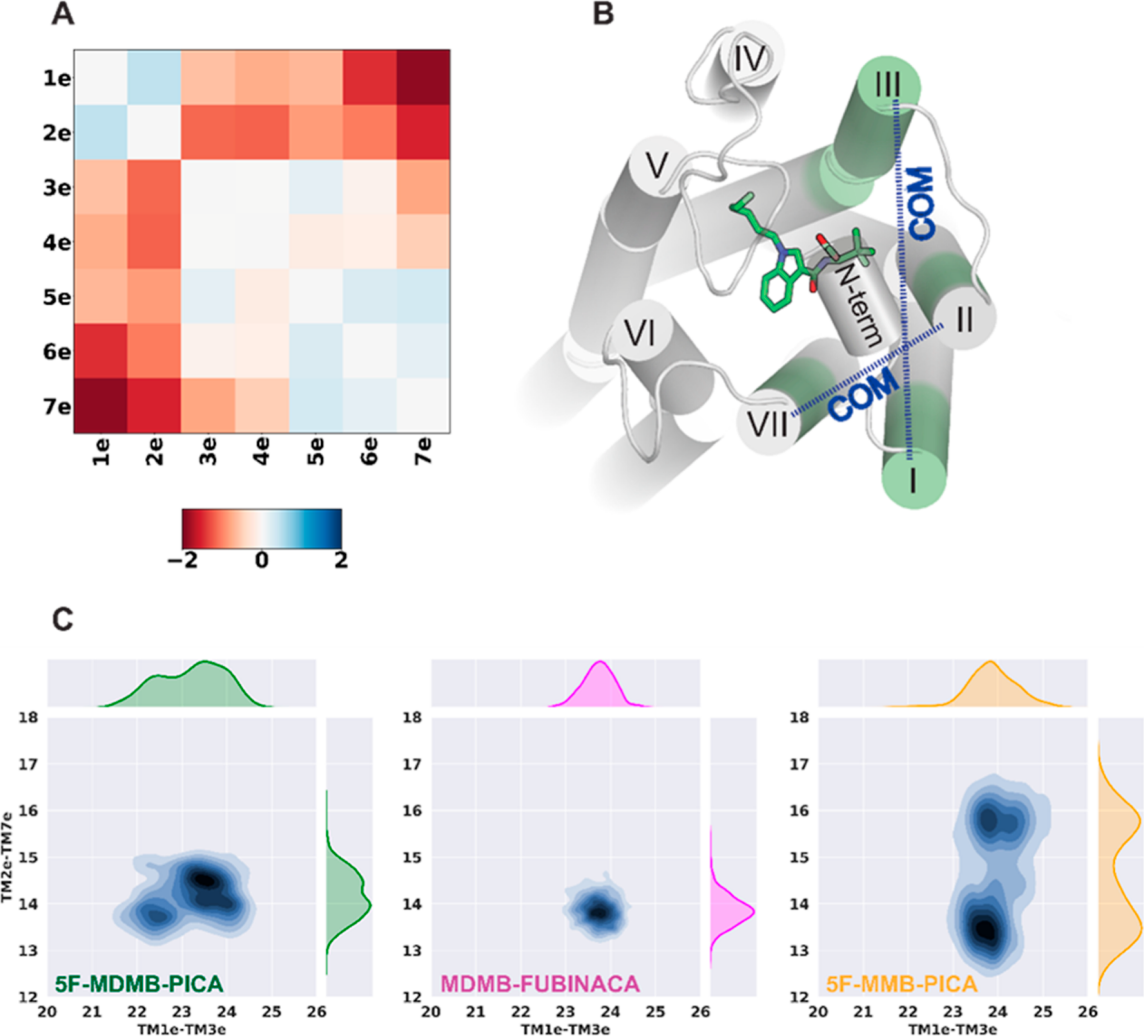
PIA-GPCR analysis and TMs’ COM distribution (A) PIA-GPCR COM results for TMs’ extracellular domain (ΔTMe(COM) = TMe(COM)_5F-MDMB-PICA_ – TMe(COM)_5F-MMB-PICA_). (B) 5F-MDMB-PICA in the binding pocket. The highlighted transmembrane subsegments are extracellular parts of TM1, TM2, TM3, and TM7 (denoted as TM1e, TM2e, TM3e, and TM7e). The blue dashed arrows show the COM distances between TM1e–TM3e and TM2e–TM7e. (C) 2D distribution of the COM distances. 5F-MMB-PICA shows two distinct distributions for TM2e–TM7e COM distance, which seems to be the result of the changes in head moiety seen for this ligand.

For our simulation results, after performing root-mean-square deviation (rmsd)-based clustering of the CB1R TM domain and conducting the PIA-GPCR analysis, we observed conformational differences in the TM1e and TM2e regions between CB1*R*/5F-MDMB-PICA and CB1R/5F-MMB-PICA (Figure 6A). Specifically, we noted that in CB1*R*/5F-MDMB-PICA, TM1e and TM2e moved inward toward the center of the ligand binding pocket, in contrast to their configuration in CB1*R*/5F-MMB-PICA. As a result of the inward movement of TM2 in CB1*R*/5F-MDMB-PICA, key residues in TM2e, such as H178^2.65^, had more frequent interactions with the head moiety of 5F-MDMB-PICA in the binding pocket (Supporting Information Table S1), thus stabilizing the bound 5F-MDMB-PICA. In comparison to the differences described above between the active and inactive CB1R structures, these trends aligned with the notion that 5F-MDMB-PICA induced the receptor to shift toward a more activated state.

To further characterize the conformational changes in this receptor region that accommodate the head groups of the SCRAs, we specifically analyzed the center-of-mass (COM) distances between two pairs of transmembrane subsegments: TM1e–TM3e and TM2e–TM7e (Figure 6B). The TM2e–TM7e distance distributions show two distinct peaks for 5F-MMB-PICA compared to 5F-MDMB-PICA and MDMB-FUBINCA (Figure 6C) due to the two conformations that the head moiety of 5F-MMB-PICA can adopt. Thus, the extra methyl group of 5F-MDMB-PICA results in a more stable conformation for its head moiety, which is associated with the changes in the dynamics of the extracellular regions of the receptor surrounding the head moiety.

### Divergence in the Allosteric Interaction Network between 5F-MDMB-PICA and 5F-MMB-PICA

**Figure 7:**
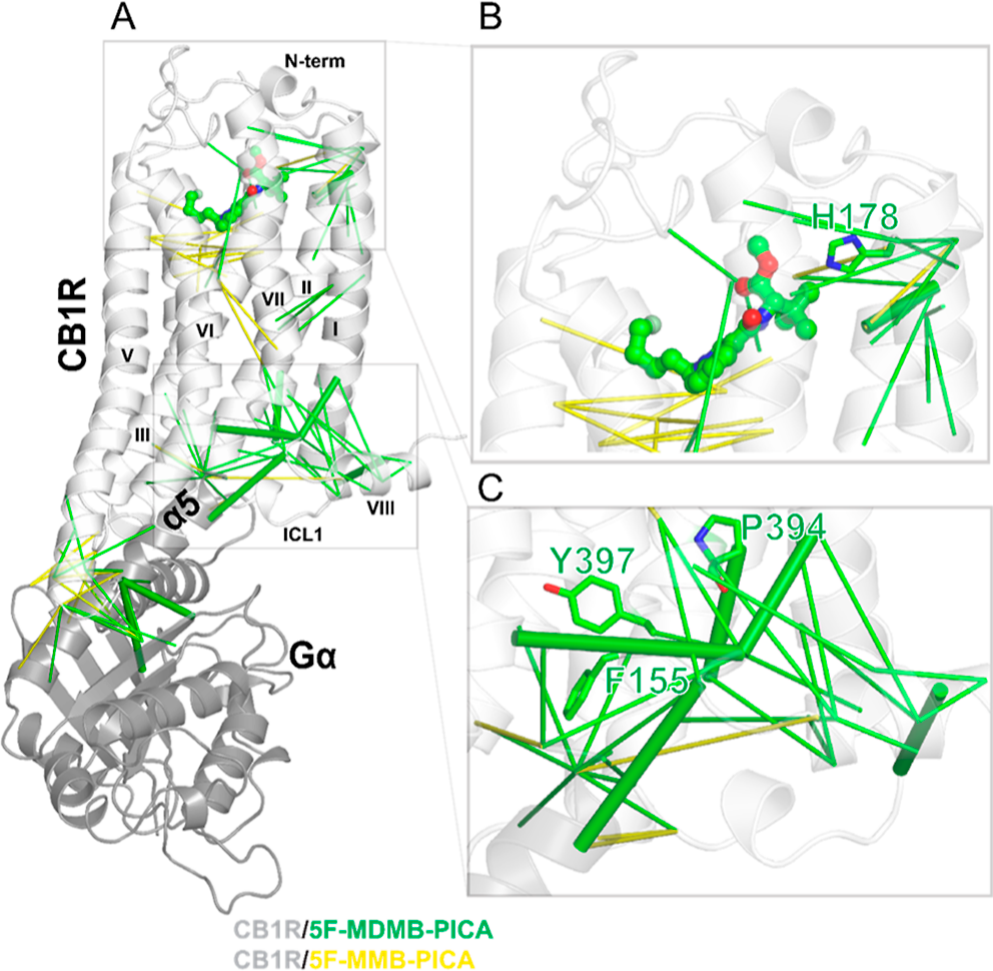
Correlation-based network pathway. (A) CB1R structure bound with 5F-MDMB-PICA with final correlated pairs mapped on the structure. The green pairs are representative pairs for 5F-MDMB-PICA and the yellow pairs are representative for 5F-MMB-PICA. There is a noticeable difference between these two ligand-signaling pathway. The CB1*R*/5F-MDMB-PICA network includes a pathway consisting of eight strongly correlated pairs (solid green lines) which connect the E133^1.49^–L399^7.55^ (TM1i–TM7i) changes to the changes at the extracellular-middle domain, F174^2.61^–D176^2.63^ (TM2m–TM2e), the intracellular domain, A160^2.47^–A398^7.54^ (TM2i–TM7i), H143^1.59^–F408^H8^ (TM1i–H8), T344^6.36^–L399^7.55^ (TM6i–TM7i), H406^H8^–R409^H8^ (H8–H8), and the changes between the intracellular domain and Gα,T313^ICL3^–S265^Gα^ (ICL3–Gα), T313^ICL3^–E318^Gα^ (ICL3–Gα), and R400^7.56^–K349^Gα^ (TM7i–Gα). E133^1.49^–L399^7.55^ (TM1i–TM7i) is at the core of these correlated pairs. There is no such pathway for CB1*R*/5F-MMB-PICA. (B) TM2e key residue H178^2.65^ contributes to the 5F-MDMB-PICA signaling pathway. (C) The TM2i residue (F155^2.42^) and TM7i NPxxY residues (P394^7.50^ and Y397^7.53^) are unique to the 5F-MDMB-PICA network pathway.

We then carried out a correlation-based network analysis (Methods) to compare how the bindings of 5F-MDMB-PICA and 5F-MMB-PICA may induce different conformational changes from the ligand binding pocket to the receptor-G protein interface. Our results identified several significant differences between the CB1*R*/5F-MDMB-PICA and CB1*R*/5F-MMB-PICA conditions, as illustrated in Figure 7.

Specifically, TMs 1, 2, and 7 are more engaged in the allosteric network for CB1*R*/5F-MDMB-PICA, while TMs 3, 4, and 5 are more involved in the CB1*R*/5F-MMB-PICA. The analysis shows that ∼45% of TM2 residues are part of the strongly correlated network for CB1*R*/5F-MDMB-PICA, while only 14% of TM2 residues are involved in the network for CB1*R*/5F-MMB-PICA. Notably, the two key residues in CB1R binding (H178^2.65^) (Figure 7B) and activation (F155^2.42^) (Figure 7C)^21^ contribute to the network for CB1*R*/5F-MDMB-PICA but not for CB1*R*/5F-MMB-PICA. Similarly, for TM7, the contribution of residues for CB1*R*/5F-MDMB-PICA and CB1*R*/5F-MMB-PICA is 44 and 15%, respectively. Among these residues, P394^7.50^ and Y397^7.53^ from the NPxxY motif are unique to the CB1*R*/5F-MDMB-PICA network (Figure 7C). As reviewed and described above, TM2 and TM7 have been shown to play critical roles in CB1R activation. Therefore, such distinct TM and residue involvements between these two conditions may be associated with the different efficacies of 5F-MDMB-PICA and 5F-MMB-PICA at CB1R.

Additionally, the number of correlated residue pairs for CB1*R*/5F-MDMB-PICA is almost twice that of CB1*R*/5F-MMB-PICA. The results showed pairs with strong correlations that connect the changes in binding pocket between the 5F-MDMB-PICA and receptor residues to other pairs (receptor–receptor, receptor-Gαi). While these residue pairs do not form a complete pathway for either CB1*R*/5F-MDMB-PICA or CB1*R*/5F-MMB-PICA, the rigid body movement of the receptor regions between them likely propagates the impact.

Thus, our results underscore the contributions of the highly conserved NPxxY motif, as well as the key residues H^2.65^ and F^2.42^^21^ in the allosteric network that may be associated with receptor activation.

## Discussion

Here, we demonstrated a substantial increase in the efficacy and potency of a 5F-pentylindole SCRA through the addition of a single methyl group to the head moiety (i.e., 5F-MDMB-PICA). This modification led to superagonism, which refers to the phenomenon where agonist efficacy surpasses that of endogenous agonists.^25^ Although “superagonism” has a clear definition, its interpretation can often be complicated when applied to assays that do not directly detect receptor activation status. The results from these assays can be confounded by signal amplification when large receptor reserves are present, which can lead to an inaccurate representation of the intrinsic efficacy of the agonist.^37^ The BRET assays utilized in this study rely on the direct coupling between a receptor and a transducer, which can effectively avoid signal amplification and detect transducer engagement or recruitment resulting from receptor activation. However, compared to the cAMP assay, the use of fusion constructs in BRET assays, which enable specificity and direct coupling, may lead to comparatively lower potencies. Nonetheless, a comparison between the test ligands and reference CP55940 ensures proper calibration of the relative potencies of SCRAs. The finding of 5F-MDMB-PICA as a superagonist at the CB1R is supported not only by our electrophysiological results but also by previous studies in cellular functional assays measuring membrane potential,^7,38^ and the cannabinoid triad behavioral assays.^39^ Notably, the presynaptic inhibition measured using field recordings in brain slices is known to be sensitive to differences in the pharmacological properties of CB1R ligands that possess an adequate “ceiling” to characterize high-efficacy agonists.

The addition of a methyl substituent, although not a dramatic structural change, is known to yield profound ligand–receptor interactions in many cases,^40,41^ as observed here with the methyl-3-methylbutanoate to methyl-3,3-dimethylbutanoate modification (i.e., MMB-to-MDMB change). Extensive interactions between the methyl-3,3-dimethylbutanoate head moiety and the TM2e region were demonstrated in the cryo-EM structure,^20^ and our computational modeling demonstrated how these interactions can significantly affect the potency and efficacy of 5F-MDMB-PICA compared to 5F-MMB-PICA. Our simulation results also indicated additional interactions of 5F-MDMB-PICA with ECL2 residues compared to 5F-MMB-PICA, which may also relate to its higher potency and efficacy. This finding aligns with previous mutagenesis work by the CB1R, which has highlighted the critical role of ECL2 in ligand binding,^20,42^ G protein coupling, and receptor trafficking.^42^ In particular, alanine scanning mutations on both N- and C-terminal regions of ECL2 have consistently resulted in more than a 10-fold increase in EC_50_ with significantly reduced *E*_max_ values, suggesting the indispensability of these residues for both ligand binding and receptor activation.^42^

Since the initial reports of illicit usage around 2010, there has been an increasing recognition of the association between the potency and efficacy of SCRAs at CB1Rs and their clinically adverse effects.^43^ Several studies have focused on assessing the pharmacological properties of the head, linker, core, and tail moieties comprising SCRAs.^44^ Consistent with the findings of our current study, previous data have also indicated that even minor structural variations within SCRAs can lead to significant efficacy changes, thereby potentially influencing clinical toxicity.^5,17,45^ Among them, 5F-MDMB-PICA, a schedule-I drug, has been identified as one of the most prevalent SCRAs in the United States, as of 2019.^46^ Fatal events have been associated with the use of this drug,^47,48^ as well as reports of notably strong depressant effects,^49^ and other toxic interactions.^50^ Our present findings suggest that even minor structural modifications in newly emerging SCRAs, particularly in relation to the presence of substituted head moieties, can strongly increase SCRA efficacy and potentially contribute to the adverse effects occurring with human consumption of these illicit compounds. Our study can be used to further our understanding of how specific alterations in CB1R ligands may contribute to their toxicity in humans and the potential life-threatening consequences of their use.

## Methods and Materials

### Cell Culture, Constructs, BRET, and Analysis

Variations of the BRET assay were performed to detect ligand-induced receptor-signaling protein coupling events. A constant amount of plasmid cDNA (15 μg) was transfected into human embryonic kidney cells 293 T (HEK-293T) using polyethylenimine (PEI; Sigma) in a 1:2 weight ratio in 10 cm plates. Cells were maintained in culture with Dulbecco’s modified Eagle’s medium (DMEM) supplemented with 10% fetal bovine serum (FBS, Atlanta), 2 mM l-glutamine (Gibco), and 1% penicillin streptomycin (Gibco) and kept in an incubator at 37 °C and 5% CO_2_. The transfected amount and ratio among the receptor and heterotrimeric G proteins were tested for the optimized dynamic range in drug-induced BRET. Experiments were performed approximately 48 h post-transfection. As reported previously,^51^ cells were collected, washed, and resuspended in phosphate-buffered saline (PBS). Approximately 200,000 cells/well were distributed in 96-well plates, and 5 μM coelenterazine H (luciferase substrate) was added to each well. One min after the addition of coelenterazine, CB1R ligands were added to each well. Two different configurations of BRET were used: (i) G_i1_-engagement and (ii) β-arrestin-2 recruitment. (i) G_i1_ protein engagement assay uses the Rluc-fused receptor and the mVenus-fused G_i1_ for a resonance energy transfer (RET) pair. Untagged Gβ1 and Gγ2constructs were cotransfected. (ii) β-Arrestin-2 recruitment uses D2R-Rluc-β-arrestin-2-Venus as a RET pair. GRK2 was cotransfected to assist in enhanced phosphorylation required for β-arrestin-2 recruitment. The donor luminescence as well as the acceptor fluorescence were always quantified for consistent expression levels across different experiments, such that there were no significant expression differences. For the fluorescence measurement, Venus was excited at 500 nm and measured at an emission wavelength of 530 nm over 1 s, using a Pherastar FSX plate reader (BMG Labtech, Cary, NC, USA). For kinetic experiments, cells were incubated at 37 °C within the Pherastar FSX plate reader (BMG Labtech, Cary, NC, USA) with BRET signal measurements taken at various time-points ranging from 2 to 46 min. The BRET signal from the same batch of cells was calculated as the ratio of the light emitted by Venus (530 nm) to that emitted by coelenterazine H (485 nm). The BRET change was defined as the BRET ratio for the corresponding drug minus the BRET ratio in the absence of the drug. *E*_max_ values are expressed as the basal subtracted BRET change and in the dose–response graphs. Data and statistical analyses were performed with Prism 9 (GraphPad Software). Bias factors between G_i1_-engagement and β-arrestin-2 were calculated as previously described.^51,52^

### Brain Slice Electrophysiology

Experiments were performed as described previously, with additional modifications.^36,51^ Brain slices were prepared from adult wild-type C57BL/6J mice (male and female) that were anesthetized with isoflurane and euthanized by decapitation. Prior to decapitation, mice were intracardially perfused with modified artificial cerebral spinal fluid (mACSF) containing (in mM): 92 NMDG, 20 HEPES, 25 glucose, 30 NaHCO_3_, 1.2 NaH_2_PO_4_, 2.5 KCl, 5 sodium ascorbate, 3 sodium pyruvate, 2 thiourea, 10 MgSO_4_, 0.5 CaCl_2_, and 300–310 mOsm, at pH 7.3–7.4. The brains were sectioned in cold mACSF, saturated with 95% O_2_ and 5% CO_2_ (carbogen), and transverse hippocampal slices (220 μm) were obtained using a vibrating tissue slicer. The slices were immediately placed in the same buffer and maintained at 32 °C for 10 min, before moving them to a holding chamber filled with carbogen-saturated ACSF (holding ACSF) containing, in mM: 92 NaCl, 20 HEPES, 25 glucose, 30 NaHCO_3_, 1.2 NaH_2_PO_4_, 2.5 KCl, 5 sodium ascorbate, 3 sodium pyruvate, 2 thiourea, 1 MgSO_4_, 2 CaCl_2_, and 300–310 mOsm, at pH 7.3–7.4. During electrophysiological recordings, slices were continuously perfused at 2 mL/min with carbogen-saturated ACSF containing (in mM): 124 NaCl, 2.5 KCl, 1.25 NaH_2_PO_4_, 1 MgCl_2_, 26 NaHCO_3_, 11 glucose, 2.4 CaCl_2_, and 300–310 mOsm, at pH 7.3–7.4. The temperature of the recording chamber was maintained at 31–32 °C. Brain slices were visualized using an upright microscope to confirm the placement of extracellular recording electrodes (3–5 MΩ), backfilled with ACSF, within the stratum radiatum of area CA1. A bipolar stimulating wire was positioned in area CA3A to activate Scc fibers and evoke fEPSPs. Extracellular recordings were made using a MultiClamp 700B amplifier (10 kHz low-pass Bessel filter) and a Digidata 1550B (20 kHz digitization) with pClamp 11 software (Molecular Devices).

As we have previously found that endogenous adenosine can disrupt CB1R-mediated inhibition of glutamate release in the hippocampus,^51^ we included the adenosine A_1_ receptor antagonist, caffeine (50 μM), in the ACSF throughout incubation and recordings.

The following numbers of slices were recorded for the drug perfusion conditions listed: 2 × 10^8^, 10^–7^, 10^–6^ M 5F-MDMB-PICA (6 slice recordings from 4 animals); 10^–8^, 10^–7^, 10^–6^ M 5F-MMB-PICA (5 slice recordings from 4 animals); 2 × 10^–8^ M 5F-MDMB-PICA + 10^–6^ M AM251 (5 slice recordings from 5 animals); 10^–6^ M 5F-MMB-PICA + 3 × 10^–6^ M AM251 (6 slice recordings from 6 animals). All data are reported as mean ± SEM. Data were analyzed in Clampex and statistically compared with Prism 9 (GraphPad Software) by a paired *t*-test.

### Molecular Dynamics Simulations

We used the cryo-EM structure of CB1R-Gαi bound with MDMB-FUBINACA (PDB 6N4B)^20^ as the main template for our modeling. The CB1R missing residues on the N-terminus (residues 104–108), ECL2 (residues 258–263), and helix 8 (H8) (residues 412–414) were added using MODELER (version 9.24) using the CB1R structure (PDB 5XRA)^19^ as the template. The structure was further processed and refined using the Protein Preparation Wizard and Ligand Preparation of Maestro (Schrödinger suite 2019-4). In addition, the residues D163^2.50^ and D213^3.49^ were protonated to their neutral forms, as assumed in the active state of rhodopsin-like GPCRs.^53^

Initial poses of 5F-MDMB-PICA and 5F-MMB-PICA were selected by analyzing the results of docking of the ligands into the binding site of the prepared CB1R model using the induced-fit docking (IFD) protocol implemented in the Schrodinger suite (Schrödinger Suite 2019-4). Using Desmond System Builder (Schrödinger suite 2019-4), CB1R models were placed into an explicit 1-palmitoyl-2-oleoyl-*sn*-glycero-3-phosphocholine lipid bilayer (POPC) with the standard membrane-protein orientation provided by the Orientation of Proteins in Membranes (OPM) database.^54^ Simple point charge (SPC) water model was used to solvate the system, charges were neutralized, and 0.15 M NaCl was added. The total system size was ∼177,000 atoms. The OPLS3e force field^55^ was used throughout this study. The initial parameters for 5F-MDMB-PICA and MDMB-FUBINACA based on the default atom typing of OPLS3e were further optimized by the force field builder (Schrödinger Suite 2019-4). The Desmond MD system (D. E. Shaw Research, New York, NY) was used for the unbiased all-atom MD simulations.

We applied a similar relaxation protocol as described previously^56^ to minimize and equilibrate the system. Briefly, the initial energy minimization was followed by equilibration with restraints on the ligand heavy atoms and protein backbone atoms. In the production runs, we used the NPT ensemble with constant temperature at 310 K maintained with Langevin dynamics, while 1 atm constant pressure was achieved by hybrid Nosé–Hoover Langevin dynamics on an anisotropic flexible periodic cell with a constant surface tension (*x*–*y* plane). In production runs, all restraints on CB1R were released. However, restraints on ***x***5 and N-terminus segments of Gα were kept for 1.2 ***x***s and then released.

### Clustering Analysis

We performed rmsd-based clustering of our aggregated MD simulation trajectories to study the protein conformational dynamics. The clustering is based on the rmsd of the backbone of the TM domain. The computed rmsd matrix was then subjected to the K-means clustering algorithm.^57^ For each ligand, we combined frames collected from all independent trajectories and then sampled 10,000 frames with replacement. Finally, we merged the sampled frames from each simulated condition and used them to calculate the average rmsd.

To estimate the number of clusters, we used the gap statistic method (1).^57^ In this technique, the change in within-cluster sum of squares error is compared with the expected value under an appropriate null distribution.
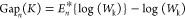
1where *E*_n_* is the expectation of log (*W*_k_), and *n* is the size of the sample from the reference distribution. *E*_n_* {log (*W*_k_)} can be estimated as following: (1) uniformly generate the reference distribution, (2) draw B number of Monte Carlo samples from the reference distribution, (3) determine the log (*W*_k_) for each sample, and (4) estimate *E*_n_*log (*W*_k_)} by an average of B copies of log (*W*_k_*). We can then calculate the gap statistic by the following two steps: (1) change the number of clusters form *k* = 1, 2, ···, *K*, cluster the observed data, and for each *k*, calculate the within-sum of squares of error (*W*_*k*_), (2) for each *k*, generate B reference data sets (*b* = 1, 2, ···, *B*) from the uniform distribution and measure the within-sum of squares of error *W*_kb_*. The gap statistic can be calculated as (eq 2)
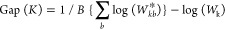
2and using , which is the simulation error in, the number of clusters is the smallest value for *k* such that Gap (*K*) ≥ Gap (*K* + 1) – *s*_*k*+1_.

Frames from selected clusters (clustered frames*) were then mapped to the original frames and saved to perform further analyses (e.g., rmsd, dihedral angles, contact frequency, protein interaction analyzer (PIA-GPCR),^58^ and correlation-based network analysis).

### Ligand Contact Analysis

We considered the heavy atoms of binding site residues and each ligand to find the residues that were within 6 Å of each ligand to calculate the contact frequency for each ligand. If any of the ligands showed 50% or more frequency for any of the binding site residues, then we tabulated the results for that residue for all studied ligands. We also calculated the differences between CB1*R*/5F-MDMB-PICA, CB1R/MDMB-FUBINACA, and CB1*R*/5F-MMB-PICA, respectively. The differences between 25% and more were highlighted.

### Conformational Analysis

The following TM subsegments were defined for the analysis of the coarse-grained interaction network of the hCB1R (PIA-GPCR). The “e”,” m”, and “i” symbols define the extracellular, middle, and intracellular sections of each TM, respectively. TM1e (residues 113^1.29^–120^1.36^), TM1m (residues 121^1.37^–129^1.45^), TM1i (residues 130^1.46–^144^1.60^), TM2e (residues 175^2.62^–179^2.66^), TM2m (residues 165^2.52^–174^2.61^), TM2i (residues 151^2.38^–164^2.51^), TM3e (residues 186^3.22^–195^3.31^), TM3m (residues 196^3.32–^202^3.38^), TM3i (residues 203^3.39^–220^3.56^), TM4e (residues 247^4.56^–253^4.62^), TM4m (residues 241^4.50^–246^4.55^), TM4i (residues 229^4.38^–240^4.49^), TM5e (residues 272^5.36^–280^5.44^), TM5m (residues 281^5.45^–288^5.52^). TM5i (residues 289^5.53^–311^5.75^), TM6e (residues 363^6.55^–368^6.60^), TM6m (residues 355^6.47^–362^6.54^), TM6i (residues 335^6.27^–354^6.46^), TM7e (residues 374^7.30^–382^7.38^), TM7m (residues 383^7.39^–390^7.46^), and TM7i (residues 391^7.47^–400^7.56^).

For PIA-GPCR side chain rotamer analysis, the residues extracted from contact frequency analysis along with the residues reported in the experimental studies^19,20^ but not identified in contact frequency results were included.

rmsd, dihedral angles, and contact frequency were calculated with VMD (version 1.9.3), PIA-GPCR with our in-house scripts, and the Cα–Cα distances for network analysis with MDAnalysis (version 1.1.1).

### Correlation-Based Network Analysis

We performed Pearson correlation analysis using the representative cluster of frames for each simulated condition to identify the allosteric pathways between the ligand binding pocket and the receptor-G protein interface. We considered three regions and included both distance and dihedral measurements for network analysis.

#### Ligand-Binding Site

For both 5F-MDMB-PICA and 5F-MMB-PICA, there are three oxygen atoms in the head moiety, two nitrogen atoms in the linker and core, respectively, and one fluorine atom in the tail. We identified the residues within 6 Å of each polar atom, and the distances between these residues and the corresponding polar atom were included in the network analysis. The dihedral angles of the ligands’ head moiety were also included in the analysis.

#### TM Domain

The Cα–Cα distances between all TM residues were calculated, and those larger than 12 Å were filtered out. The dihedral angles of the conserved motifs playing critical roles in GPCR activation (C^6.47^W^6.48^x^6.49^P^6.50^, D^3.49^R^3.50^Y^3.51^, P^5.50^I^3.40^F^6.44^, and N^7.49^P^7.50^x^7.51^x^7.52^Y^7.53^) were also measured and included in the analyses.

#### CB1R-Gαi Interface

The Cα–Cα distances between the receptor and the Gα residues were calculated, and those larger than 12 Å were filtered out.

The Pearson correlation coefficient (*r*) was calculated for all distance/dihedral pairs. We then applied three filtering criteria to the Pearson correlation coefficient matrix: (1) only pairs with *r* ≥ 0.85 were kept, (2) for a correlated pair of distances, *d*_*i*_ = *X*_*i*_ – *Y*_*i*_ [distance (*i*) and *d*_*j*_ = *X*_*j*_ – *Y*_*j*_ (distance *j*)], where *X* and *Y* are the residue numbers, if {(|(*X*_*i*_ – *X*_*j*_)| or |(*Y*_*i*_ – *Y*_*j*_)|) ≥ 8}, then this correlated pair was kept, and (3) for the correlated pairs that share one common distance (*d*_*i*_), while the other distances (*d*_*j*_) are formed by residues from the same pair of TM subsegments (see subsegment definitions in conformational analysis above), only the correlated pair with the maximum difference between residue numbers (|(*X*_*i*_ – *X*_*j*_)| or |(*Y*_*i*_ – *Y*_*j*_)|) was kept.

The clustering and network analyses were repeated 10 times (bootstrap sampling with replacement). The commo-correlated pairs for all 10 bootstrap samplings were extracted for each ligand and mapped to the structure for visualization.

## Supporting Information Available

Contact frequency and side chain dihedral angles for CB1R bound with 5F-MDMB-PICA, 5F-MMB-PICA, and MDMB-FUBINACA; chemical structures; G_i1_ engagement BRET; β-arrestin 2 recruitment BRET; brain slice electrophysiology; 2D structures of 5F-MMB-PICA, 5F-MDMB-PICA, and MDMB-FUBINACA; and PIA-GPCR analysis of the experimentally determined structures (PDF)
