# Supplementary material for: Subtle Structural Modification of a Synthetic Cannabinoid Receptor Agonist Drastically Increases its Efficacy at the CB1 Receptor
Source: ACS Chem Neurosci. 2023 Oct 17;14(21):3928–40. doi: 10.1021/acschemneuro.3c00530 (PMC10623572; doi:10.1021/acschemneuro.3c00530)

A subtle structural modification of a synthetic cannabinoid receptor agonist drastically increases its efficacy at the CB1 receptor

Hideaki Yano[*,#,1], Rezvan Chitsazi[#,2], Christopher Lucaj[1], Phuong Tran[2], Alexander F. Hoffman[3], Michael H. Baumann[4], Carl R. Lupica[3], Lei Shi[*,2]

[1]Department of Pharmaceutical Sciences, School of Pharmacy and Pharmaceutical Sciences, Bouvé College of Health Sciences, Center for Drug Discovery, Northeastern University, Boston Massachusetts 02115

[2]Computational Chemistry and Molecular Biophysics Section, [3]Electrophysiology Research Section, and [4]Designer Drug Research Unit, Intramural Research Program, National Institute on Drug Abuse, National Institutes of Health, Baltimore, Maryland 21224

#These authors contributed equally

*Co-corresponding authors

Keywords: synthetic cannabinoids, cannabinoid receptor 1, bioluminescence resonance energy transfer, molecular dynamics.

SUPPORTING INFORMATION

Supplementary Table 1. Contact frequency and sidechain dihedral angles for CB1R bound with 5F-MDMB-PICA (DP), 5F-MMB-PICA (MP), and MDMB-FUBINACA (DF).

Residues are shown as one letter with residue numbers and Ballesteros-Weinstein numbering for GPCRs in parenthesis. We used 25% difference as the threshold to identify residues having divergent contact frequencies and 60-degree difference as the threshold to identify the residues having divergent sidechain rotamers (or the distributions).

	Contact Frequency					PIA-GPCR (Sidechain dihedral angles)				
Residue	DP	MP	DF	(DP – MP)	(DF – MP)	DP	MP	DF	(DP – MP)	(DF – MP)
I169 (2.56)	43.6	93.4	66.0	-49.7	-27.3	275.3	279.8	286.9	4.5	7.1
F170 (2.57)	99.9	100.0	100.0	0.0	0.0	180.7	180.3	178.9	0.4	1.4
V171 (2.58)	0.0	66.0	20.5	-66.0	-45.5	233.0	203.4	212.9	29.6	9.5
S173 (2.60)	99.9	100.0	100.0	0.0	0.0	133.2	205.5	111.8	-72.3	-93.7
F174 (2.61)	99.9	98.6	92.7	1.3	6.0	200.1	185.3	190.7	14.8	5.4
F177 (2.64)	99.9	99.9	100.0	0.0	0.0	187.2	247.8	228.2	60.6	19.6
H178 (2.65)	95.4	46.3	100.0	49.1	53.6	276.1	285.0	258.6	8.9	26.4
F189 (3.25)	89.6	96.9	99.8	7.3	2.8	178.5	180.7	179.6	2.2	1.1

K192 (3.28)	67.6	94.2	90.1	26.6	4.1	281.9	286.1	288.1	4.2	2.0
L193 (3.29)	99.9	100.0	100.0	0.0	0.0	191.9	181.0	185.1	10.9	4.1
G194 (3.30)	0.0	60.4	0.0	-60.4	-60.4	0.0	0.0	0.0	0.0	0.0
V196 (3.32)	99.9	100.0	100.0	0.0	0.0	178.8	169.3	180.5	9.5	11.2
T197 (3.33)	99.9	100.0	100.0	0.0	0.0	308.3	306.6	307.4	1.7	0.8
F200 (3.36)	99.9	99.6	99.5	0.3	0.1	278.4	274.3	282.2	4.1	7.9
I267 (ECL2)	76.0	28.5	55.3	47.5	26.8	267.8	206.9	262.3	60.9	55.4
F268 (ECL2)	99.9	100.0	100.0	0.0	0.0	289.9	279.8	288.3	10.1	8.5
P269 (ECL2)	60.4	0.0	10.1	60.4	10.1	166.5	89.4	207.8	77.1	118.4
H270 (ECL2)	43.3	16.8	50.0	26.5	33.2	221.1	240.2	150.1	19.1	90.1
I271 (ECL2)	98.3	82.8	99.0	15.5	16.2	131.8	145.0	130.0	13.2	15.0
D272 (5.36)	29.6	11.5	53.1	18.2	41.6	183.1	183.5	183.1	0.4	0.4
Y275 (5.39)	99.8	88.4	100.0	11.3	11.5	178.5	179.9	180.8	1.4	0.9
L276 (5.40)	99.8	96.0	100.0	3.9	4.0	217.3	254.5	218.8	37.2	35.7
W279 (5.43)	99.8	100.0	100.0	0.2	0.0	185.9	185.8	186.4	0.1	0.6
W356 (6.48)	62.2	57.3	69.3	5.0	12.0	293.5	293.5	295.4	0.0	1.9

L359 (6.51)	99.4	99.4	100.0	0.0	0.5	227.2	250.2	238.3	23.0	11.9
M363 (6.55)	99.9	99.4	99.9	0.5	0.5	287.1	278.0	289.4	9.1	11.4
F379 (7.35)	99.5	97.6	98.4	1.9	0.8	178.4	181.5	181.9	3.1	0.4
C382 (7.38)	31.0	51.4	30.6	20.4	20.8	287.3	290.2	287.6	2.9	2.6
S383 (7.39)	99.9	98.5	100.0	1.4	1.5	182.8	137.4	213.8	45.4	76.4
C386 (7.42)	98.9	94.7	99.6	4.1	4.9	286.2	286.2	295.0	0.0	8.8

Supplementary Figure 1. Chemical structures

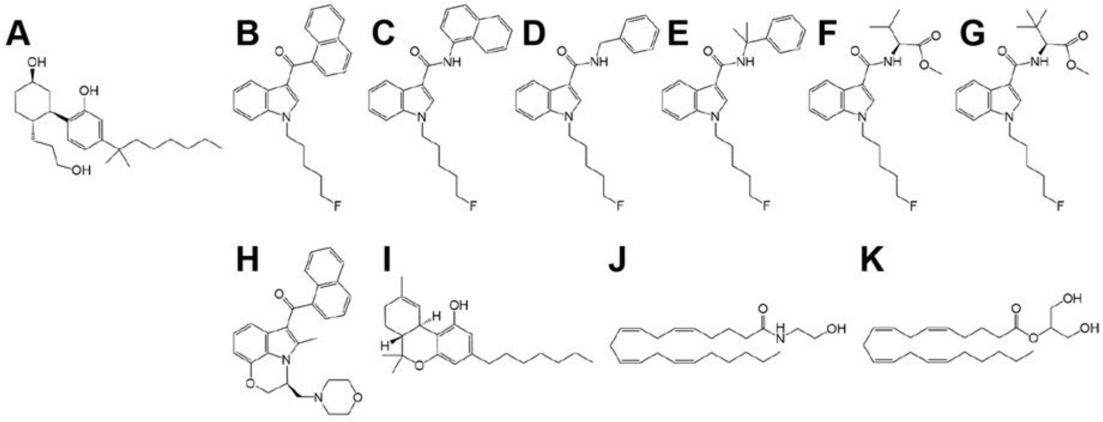

A. CP55940, B. AM2201, C. 5F-NNEI, D. 5F-SDB-006, E. 5F-CUMYL-PICA, F. 5F-MMB-PICA, G. 5F-MDMB-PICA, H. WIN55-212-2, I. Δ^9-THC, J. AEA, K. 2-AG

Supplementary Figure 2. G_{i1} engagement BRET

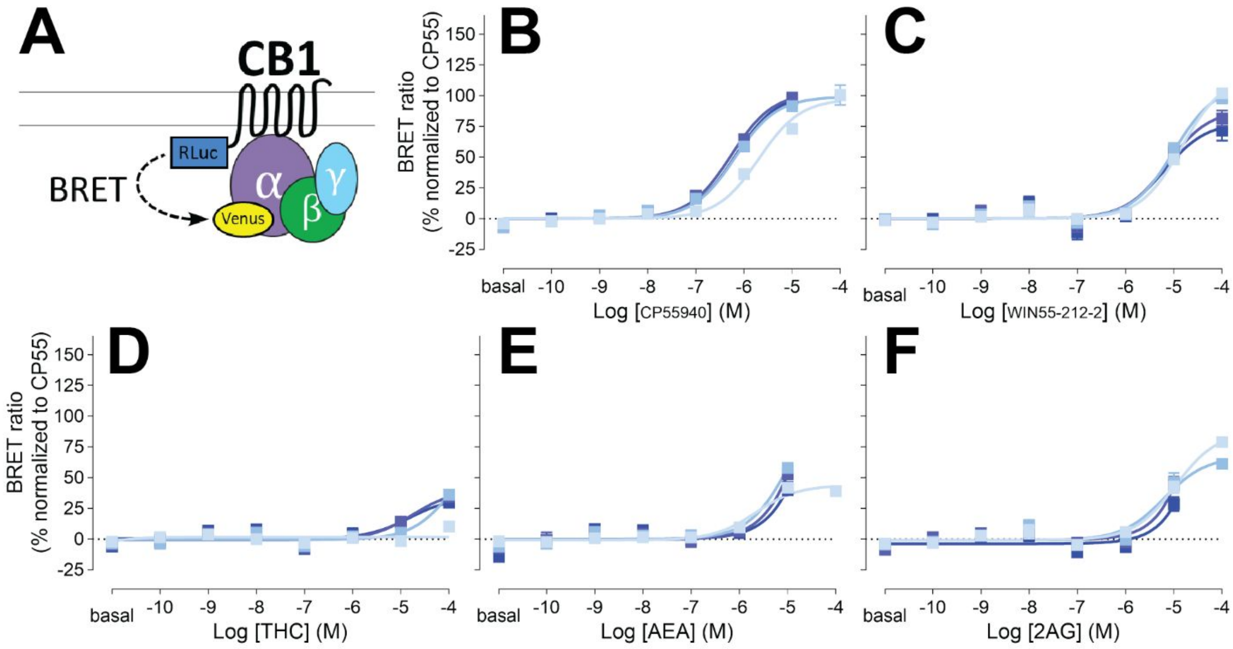

Drug-induced engagement BRET between CB1R-Rluc and Gi1-Venus (A) measured in response to CP55940 (B), WIN55-212-2 (C), Δ^9-THC (D), anandamide (E), 2-arachidonoylglycerol (F) at various time points (2, 16, 30, 44 min light to dark blue). Concentration-response curves are plotted as a percentage of maximal response by CP55940 at each time point and presented as means ± SEM of $n \geq 3$ independent experiments.

Supplementary Figure 3. β-arrestin 2 recruitment BRET

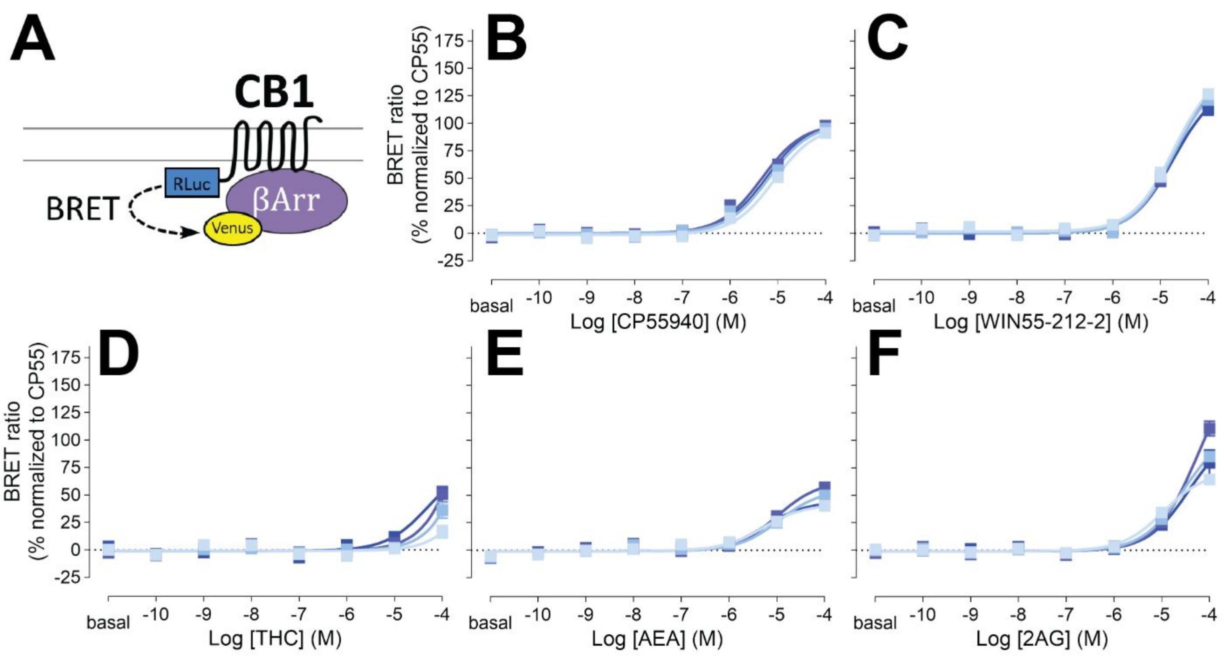

Drug-induced recruitment BRET between CB1R-Rluc and β-arrestin 2-Venus (A) measured in response to CP55940 (B), WIN55-212-2 (C), Δ^9-THC (D), anandamide (E), 2-arachidonoylglycerol (F) at various time points (2, 16, 30, 44 min light to dark blue). Concentration-response curves are plotted as a percentage of maximal response by CP55940 at each time point and presented as means ± SEM of *n* ≥ 3 independent experiments.

Supplementary Figure 4. Brain slice electrophysiology

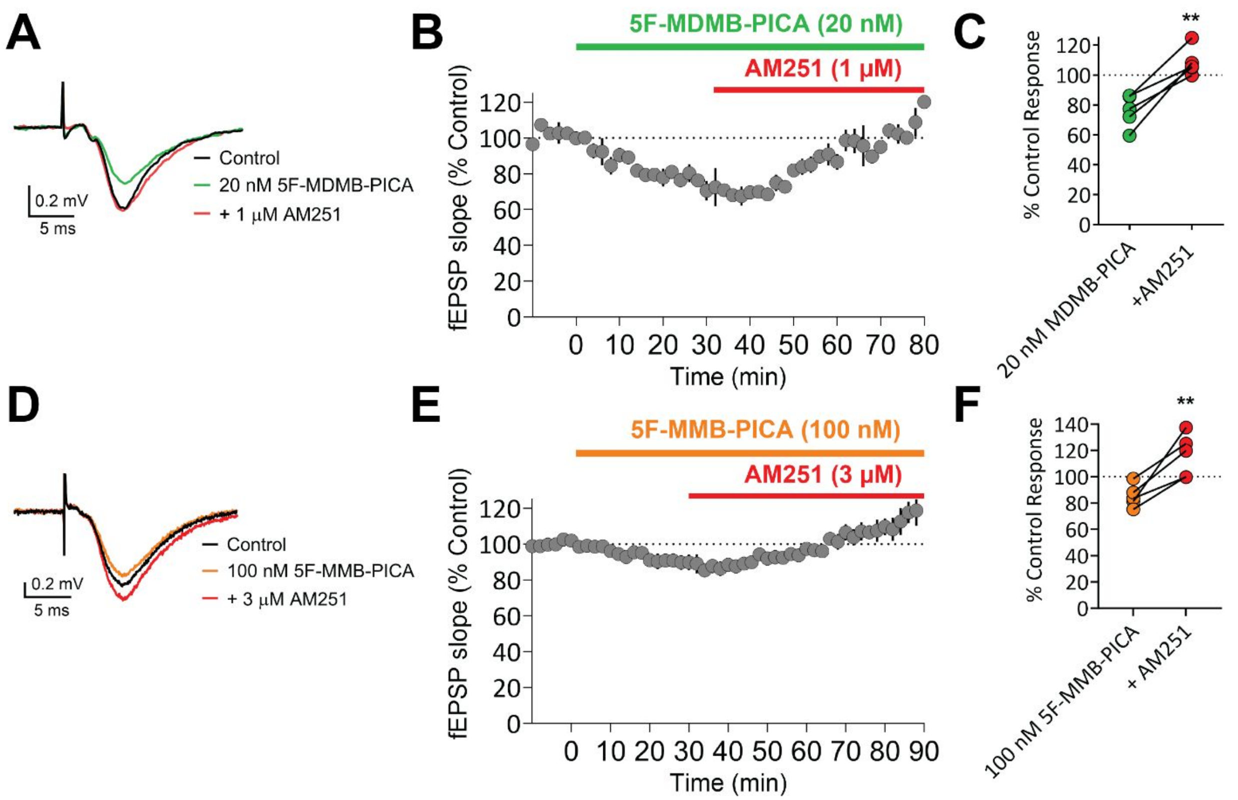

Reversal of hippocampal glutamate release inhibition by CB1R selective antagonist. (A,D) Representative averaged traces for 5F-MDMB-PICA and its reversal by AM251 (A) and 5F-MMB-PICA and its reversal by AM251 (D), (B,E) Summary time course of recordings ($n \geq 3$ recordings) demonstrating the effects of 5F-MDMB-PICA (B), 5F-MMB-PICA (E), and their reversal. (C,F) Individual recording result of reversal effect by AM251 for 5F-MDMB-PICA (C) and 5F-MMB-PICA (F). Data points are presented as means ± SEM. Paired t-test shows **p < 0.01.

Supplementary Figure 5. 2D structures of 5F-MMB-PICA, 5F-MDMB-PICA, and MDMB-FUBINACA.

The head moiety dihedral angles ($\boldsymbol{x}1$ and $\boldsymbol{x}2$) are shown as arrows which are rotation around N-C and C-C, respectively. The same dihedral angle definitions apply to 5F-MDMB-PICA and MDMB-FUBINACA.

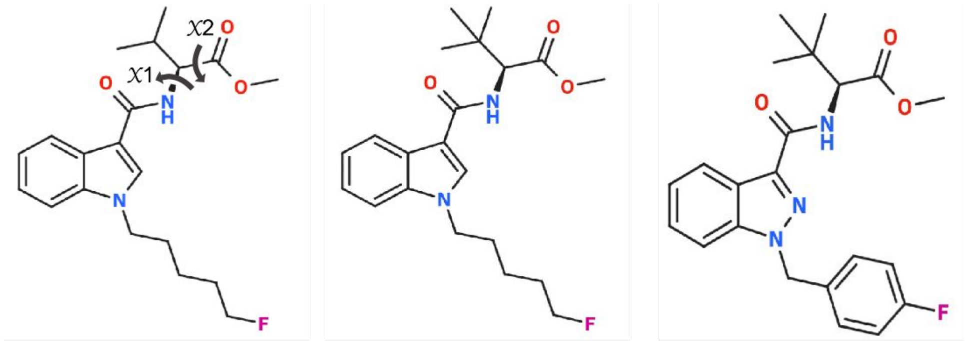

5F-MMB-PICA 5F-MDMB-PICA MDMB-FUBINACA

Supplementary Figure 6. PIA-GPCR analysis of the experimental determined structures

The comparisons are between active hCB1-Gi state (PDBs: 6N4B, 6KPG, and 7WV9) and inactive hCB1 (PDB: 5U09 and 5TGZ). (A) The COM distances between extracellular domain of TMs denoted as (1-7)e. (B) The COM distances between the intracellular domain of TMs denoted as (1-7)i. Among the three active structures, 6N4B has the larger differences from the inactive structures on the extracellular side, which is represented by the larger inward movement of TM2e and outward movement of TM6e. On the intracellular side, due to the conforming effect of binding to the Gi protein, no drastic difference was observed.

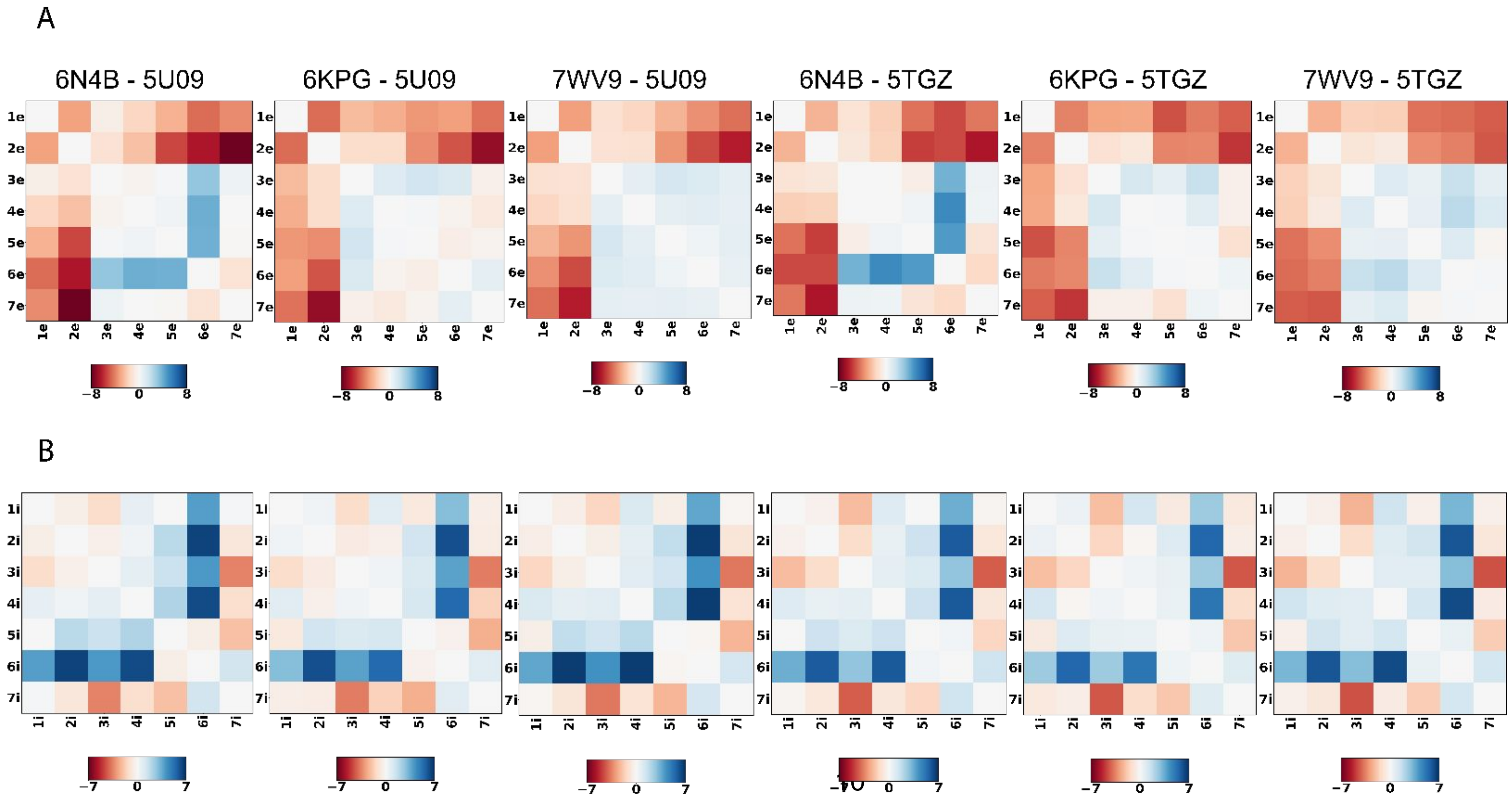

Supplement: Supplementary file 1 — cn3c00530_si_001.pdf [file cn3c00530_si_001.pdf]
